# Diagnosis and Dentists' Treatment Preferences for Vestibular Enamel Defects—A Cross‐Sectional Survey

**DOI:** 10.1111/jerd.70217

**Published:** 2026-06-22

**Authors:** Basel Kharbot, Marcus Cebula, Vinay Pitchika, Falk Schwendicke, Susanne Effenberger

**Affiliations:** ^1^ Department of Operative, Preventive and Pediatric Dentistry, Center for Dental and Craniofacial Sciences Charité—Universitätsmedizin Berlin Berlin Germany; ^2^ Clinical Research, DMG Hamburg Germany; ^3^ Department of Conservative Dentistry, Periodontology and Digital Dentistry LMU University Hospital Munich Germany

**Keywords:** DDEs, diagnostic accuracy, enamel defects, enamel opacities

## Abstract

**Objectives:**

Vestibular enamel defects, including WSL, MIH, or fluorosis, can impair esthetics and patient well‐being, making accurate diagnosis essential for selecting appropriate treatment strategies. However, distinguishing these based solely on clinical images—an increasingly common scenario in digital communication—can be challenging. This pilot study aimed to assess dentists' diagnosis accuracy and confidence in assessing clinical images of vestibular enamel defects, and how the diagnosis influenced treatment choices.

**Materials and Methods:**

A cross‐sectional survey was distributed primarily to dentists from Europe and South America. It included demographic questions and 10 clinical images (two showing WSL, three fluorosis, four MIH, and one MIH/enamel hypoplasia combined). Reference diagnoses were established based on consensus among three specialists. Participants were asked to diagnose each image, select their preferred treatment options, and rate their diagnostic and therapeutic confidence using a 5‐point Likert scale.

**Results:**

A total of 168 dentists completed the survey. Overall, diagnostic accuracy was 52%, with the highest for fluorosis (66%) and the lowest for the mixed case (33%). Most participants correctly diagnosed 3–8 of 10 cases. Conservative treatment options were chosen primarily, with resin infiltration selected in 70% of responses regardless of diagnosis. Diagnostic confidence was reported in 72% of cases.

**Conclusion:**

The diagnostic accuracy of vestibular enamel defects based on clinical images was limited, and overconfidence was frequent. Dentists preferred conservative treatment strategies, though their choices were influenced by diagnostic accuracy.

## 1 Introduction

Vestibular enamel defects, including carious white‐spot lesions (WSL), molar‐incisor hypomineralization (MIH), and dental fluorosis, are either acquired or developmental anomalies that can substantially affect both the function and esthetic appearance of teeth [1, 2, 3]. Beyond their clinical implications, these conditions can greatly impact an individual's self‐perception and self‐esteem, as an attractive smile is associated with confidence, well‐being, and perceived intelligence. Conversely, dissatisfaction with their dental appearance may cause reduced confidence and increased self‐consciousness in daily interactions. Children, adolescents, and young adults may be particularly affected, as this period is critical for personality development and social engagement [4, 5, 6].

The esthetic management of vestibular enamel defects has demonstrated favorable clinical results, along with considerable improvements in oral health‐related quality of life (OHRQoL) and patient satisfaction [7, 8, 9, 10]. Although a variety of treatment options exist, it is generally recommended to opt for the most conservative approaches feasible in order to preserve hard dental tissue, a consideration particularly crucial in younger patients. This has resulted in increased awareness of the significance of minimally invasive and targeted therapeutic strategies [7, 11, 12, 13, 14, 15, 16].

Importantly, although some therapeutic approaches may be suitable for a wide range of cases, the appropriate treatment strategy can vary substantially between cases due to differences in etiology, defect characteristics, and the patient's treatment goals. For instance, noninvasive remineralization strategies are commonly used to manage WSL, yet they are less appropriate for fluorosis, which is itself a consequence of excessive fluoride intake. Mild fluorosis often does not require intervention, as it is frequently not associated with reduced OHRQoL and thus not a concern for the patient [17]. In contrast, more severe fluorosis cases and MIH in general may benefit significantly from bleaching (to remove dark discolorations), whereas resin infiltration can effectively mask a broad range of whitish vestibular defects [10, 11, 18, 19]. To date, there is a lack of detailed, differentiating data on dentists' treatment preferences for the various types of vestibular enamel defects, with the existing literature suggesting that these preferences vary according to the type of defect [20].

Consequently, accurate diagnosis is vital for selecting the most suitable treatment approach. A key parameter in this process is the clinical presentation of the defect. MIH generally manifests as well‐demarcated, whitish‐yellow to yellow‐brown opacities on buccal surfaces of the central incisors. However, lateral incisors and canines can also be affected [21, 22]. In contrast, fluorosis is characterized by white‐to‐brown spots or streaks with diffuse margins, with symmetrically affected teeth, most often located in the central or incisal regions of the incisors [3, 23]. However, diffuse enamel opacities or dysmineralizations with fluorosis‐like clinical presentations may have different etiologies. In the present study, the term fluorosis was used because of its wider use among clinicians and in the literature. WSLs manifest as chalky white opacities in areas prone to plaque accumulation, such as gingival margins cervically or around orthodontic brackets [24, 25]. More severe cases across these conditions may additionally display structural defects or enamel breakdown. Mixed defects may incorporate multiple clinical features, including those quantitative and qualitative defects mentioned above. They may also involve enamel hypoplasia, which results from alterations during enamel formation. Such defects can be caused by various factors, including local causes (e.g., trauma), genetic predispositions, or environmental influences, leading to either localized or widespread clinical manifestations [26, 27].

While numerous studies have examined diagnostic capabilities, they have typically included some form of preliminary training, concentrated on assessing the applicability of specific diagnostic indices, or limited their scope to individual defects [28, 29, 30, 31, 32, 33]. To the best of our knowledge, there is a lack of data concerning the accuracy of dentists in identifying various types of vestibular enamel defects solely through clinical images, an approach that closely resembles daily clinical practice, as well as how their diagnostic precision influences their treatment decisions in the absence of prior, study‐specific training or guidance [20].

The objective of this pilot cross‐sectional survey was therefore to evaluate the diagnostic accuracy of dentists in assessing vestibular enamel defects solely from frontal images, as well as to investigate their confidence in their diagnostic abilities. In addition, we aimed to determine whether dentists' treatment recommendations were influenced by their diagnostic accuracy.

## 2 Materials and Methods

This cross‐sectional survey received approval from the Institutional Ethical Review Board of Charité University on June 16, 2022 (EA4/069/22). A convenience sample of dentists from Europe and South America was invited to participate through study clubs and professional networks between November 2022 and December 2023.

This survey was designed in Microsoft Forms and pretested by three dental professionals to ensure clarity and comprehensibility; modifications were made based on their feedback. Distribution was conducted via email. Data collection was conducted anonymously.

The survey consisted of two main sections: (1) demographic information—age, gender, years of professional experience, area of specialization, educational level, work environment, and current geographical location—and (2) an assessment of 10 clinical cases: two with WSL, three with fluorosis, four with MIH, and one with a mixed lesion (MIH and enamel hypoplasia). Each case was represented by a frontal view image (Figure 1). Participants were asked to diagnose each case, select one or more treatment options, and rate their confidence in both their diagnosis and treatment decisions using a 5‐point Likert scale. Reference diagnoses were determined based on clinical examination and consensus among three specialists, each with at least 5 years of experience in managing vestibular enamel defects.

**FIGURE 1:**
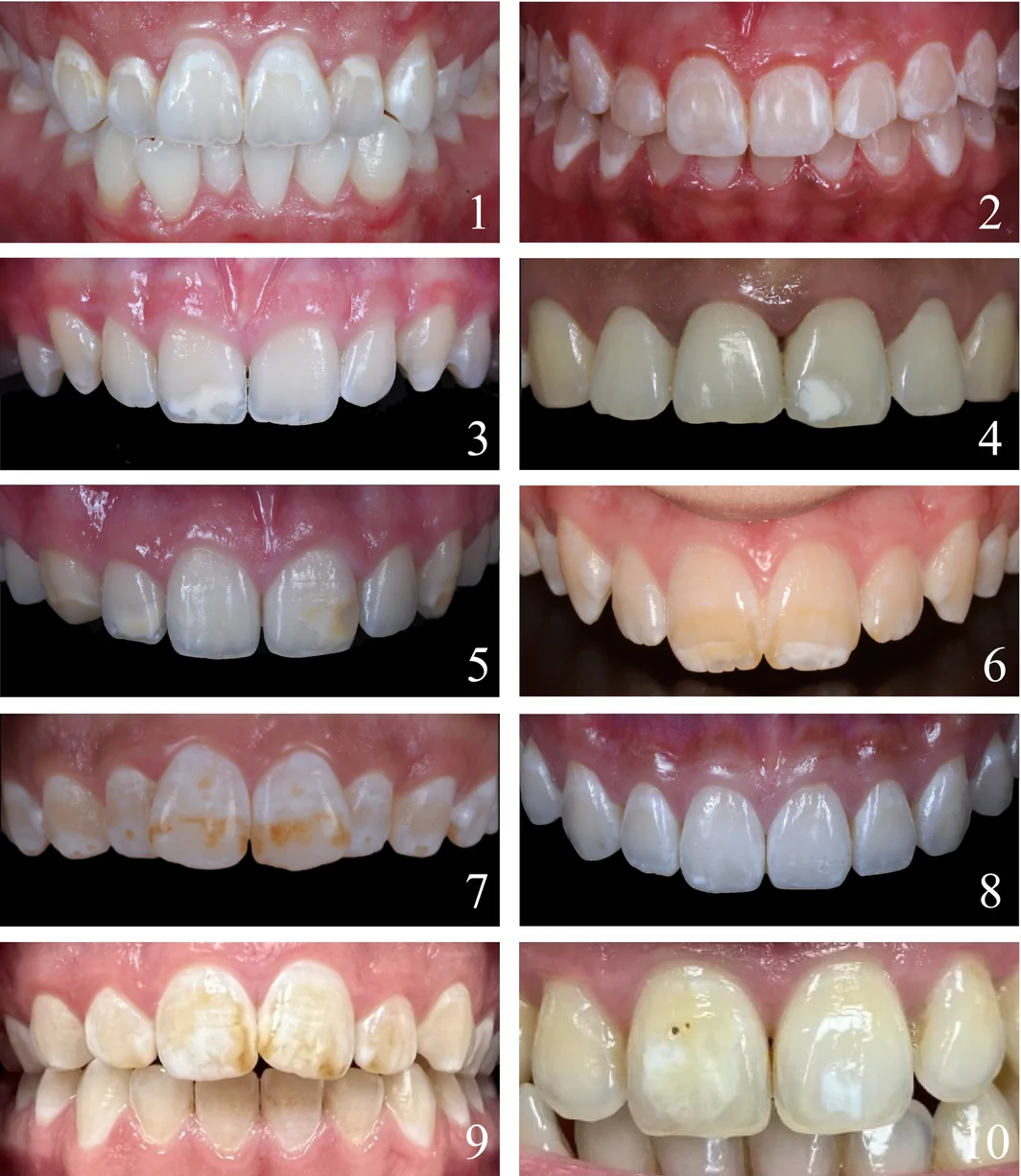
Clinical cases presented in the questionnaire. The correct diagnoses, determined by majority vote, were as follows: WSL (1–2); MIH (3–6); fluorosis (7–9); mixed (10).

Survey responses were exported to Microsoft Excel 365 and analyzed using Stata MP 19 (StataCorp. 2025 College Station, TX). Participants' reported areas of specialization were categorized according to the predefined groups shown in Table 1. Due to their functional equivalence, the work environment categories “employed at private practice” and “employed at clinic” were combined into a single category (“employed at practice or clinic”). Likewise, the education‐level response options “doctorate,” “PhD,” and “Professor” were merged into one category (“Doctorate/PhD”). A binary variable for diagnostic accuracy was generated by comparing each participant's diagnosis against the reference (gold‐standard) diagnosis. Categorical data were summarized as proportions. Diagnostic accuracy was evaluated using sensitivity, specificity, positive predictive value, and negative predictive value for each diagnostic category, and overall agreement was assessed using Cohen's kappa. Exact binomial tests were used to determine whether observed diagnostic accuracy exceeded the 25% expected by chance alone, both overall and stratified by the reference diagnosis. Confidence in diagnosis and treatment was categorized by collapsing the 5‐point Likert scale into two groups: responses in the first two points were classified as “confident”, while the remaining three points were classified as “not confident”. Differences in diagnostic difficulty across the 10 clinical cases were assessed using mixed‐effects logistic regression with case as a fixed effect and a random intercept for participant; within‐group comparisons were performed for diagnostic categories containing more than one case, with Bonferroni correction for multiple pairwise comparisons. Mixed‐effects logistic regression with a random intercept for participant was used to assess factors associated with correct diagnosis, with sex, age, work region, years of clinical experience, and employment status as fixed effects, stratified by the reference diagnosis. Standard logistic regression models were then used to evaluate the influence of diagnostic accuracy, treatment confidence, sex, age, work region, years of clinical experience, and employment status on each treatment preference (no treatment, noninvasive treatment, bleaching, microinvasive treatment, composite resin restoration, and veneer), stratified by the reference diagnosis. Crowns were excluded from models because very few participants recommended this treatment option, resulting in a very small number of observations and convergence issues during model estimation. Odds ratios with 95% confidence intervals were reported for all regression models. A significance level of *p* < 0.05 was applied for all tests.

**TABLE 1:** Demographics of participants (*n* = 168).

Item		Participants: count (%)	
Male/female		70m (42%)/98f (58%)	
Location^a^	Europe	98	(58%)
	South America	64	(38%)
	North America	3	(2%)
	Middle East and Africa	3	(2%)
Experience	< 5 years	32	(19%)
	5–10 years	49	(29%)
	11–20 years	35	(21%)
	21–30 years	32	(19%)
	> 30 years	20	(12%)
Employment	Employed at praxis or clinic	40	(24%)
	Employed at university	53	(32%)
	Self‐employed	46	(27%)
	Other	28	(17%)
Highest education level	Graduation	39	(23%)
	MSc	32	(19%)
	Specialization course	22	(13%)
	Doctorate/PhD	75	(45%)
Specialization^b^	Operative dentistry	58	(32%)
	Pediatric dentistry	33	(18%)
	General dentistry	30	(16%)
	Prosthodontics	15	(8%)
	Oral surgery	11	(6%)
	Periodontology	10	(5%)
	Orthodontics	8	(4%)
	Public health	2	(1%)
	Special needs	2	(1%)
	Hygienist	2	(1%)
	NA (not available)	14	(8%)

^a^ Participants from Europe were primarily from Germany (53), Portugal (16), and Italy (8), while participants from South America were almost exclusively from Brazil (63). Numbers indicate participant counts.

^b^ Participants were able to indicate more than one specialization. Consequently, the total count of specializations (185) exceeds the number of participants (168).

## 3 Results

A total of 856 dentists were invited to participate, of whom 168 completed the survey, resulting in a response rate of 20%. Most participants were female (58%) and primarily based in Europe (58%) and South America (38%) (Table 1). The distribution of work experience and job roles was balanced. The largest group had 5 to 10 years of experience (29%), while those with over 30 years made up the smallest segment (12%). Approximately half of the respondents (45%) held a PhD or Doctorate, with the highest participation among university employees (32%). The most common specialty was operative dentistry (32%), followed by pediatric dentistry (18%) and general dentistry (16%).

Out of 1680 total diagnostic attempts (168 participants × 10 cases), 885 were correct, which is an observed accuracy of 52%. If diagnoses were purely random guessing among the four options, one would expect an accuracy of 25%. The two‐sided *p* value confirmed that overall diagnostic accuracy was significantly better than chance (*p* < 0.001, Figure 2). When stratified by reference diagnosis (Table 2), accuracy was highest for fluorosis (66%, 335/504; *p* < 0.001), followed by WSL (50%, 167/336; *p* < 0.001) and MIH (49%, 328/672; *p* < 0.001). The mixed lesion had the lowest accuracy (33%, 55/168), although it remained significantly above chance (*p* = 0.026).

**FIGURE 2:**
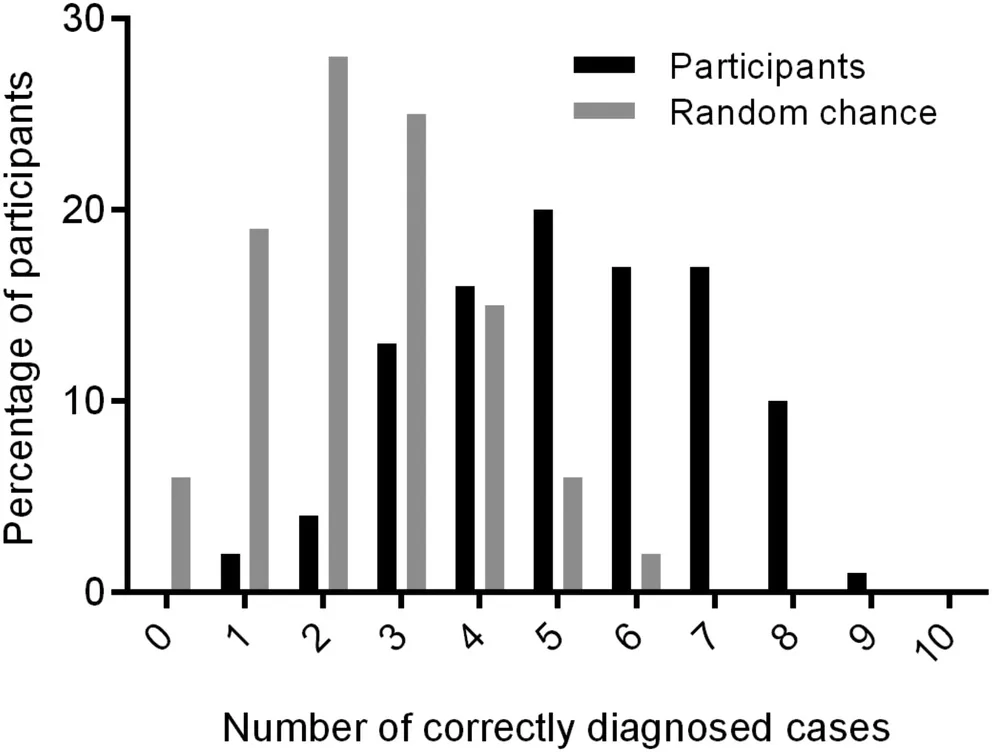
Distribution of participants' diagnostic accuracy, showing the percentage of participants who correctly diagnosed between 0 and 10 out of 10 cases. The “Random chance” curve represents a binomial distribution that models diagnostic accuracy when participants guess on all cases (with a 0.25 probability of guessing a case correctly, given 4 possible diagnoses to choose from). Participants' diagnostic accuracy was significantly different from chance (*p* < 0.001).

**TABLE 2:** Diagnostic accuracy.

Dentists' diagnosis	Reference diagnosis			
	WSL	Fluorosis	MIH	Mixed
WSL	**50**	2	1	5
Fluorosis	18	**66**	28	12
MIH	5	11	**49**	50
Mixed	26	21	22	**33**

*Note:* Values represent the percentage (%) of cases diagnosed with each listed condition.

Bold numbers indicate the percentage of correct diagnoses.

Statistically significant differences between correct diagnosis and chance are highlighted in gray (*p* < 0.05).

Misclassification patterns differed by condition. Misdiagnoses as WSL or MIH were generally uncommon, except for the mixed case, which was diagnosed as MIH in 50% of instances. Given that this case truly represented both MIH and enamel hypoplasia, these findings should be interpreted with caution. In contrast, misdiagnosis as fluorosis occurred more frequently, particularly for MIH cases (28%). Additionally, nearly one quarter of all responses were incorrectly categorized as “mixed” (Table 2). Diagnostic performance metrics further highlighted differences between conditions (Table S1). Specificity was highest for WSL (98%), reflecting a low false‐positive rate, whereas fluorosis and mixed lesions showed more moderate specificity (both 77%). Positive predictive values were highest for WSL (86%) and MIH (68%), but notably low for mixed lesions (14%), indicating substantial overclassification of this category. Negative predictive values were consistently high across diagnoses, especially for mixed lesions (91%), suggesting that participants were generally reliable at ruling out conditions when they were absent. Most participants correctly diagnosed between 3 and 8 of the 10 cases (Figure 2).

Diagnostic difficulty varied significantly across the 10 clinical cases (*χ* ^2^ = 160, *p* < 0.001; see Figure 1 for cases), with case‐level accuracy ranging from 19% (case 6, MIH) to 71% (case 7, fluorosis). Within each diagnostic category, significant differences in difficulty were also observed. Among the two WSL cases, case 1 was significantly easier than case 2 (59% vs. 41%; OR 2.6, *p* < 0.001). Among the three fluorosis cases, accuracy differed significantly (*p* = 0.008), with cases 7 and 8 (both 71%) being easier than case 9 (57%). Among the four MIH cases, difficulty varied substantially (*p* < 0.001), driven primarily by case 6 (19%), which was markedly harder than cases 3, 4, and 5 (64%, 57%, and 55%, respectively; OR 0.14 for case 6 vs. case 5). The mixed lesion category contained a single case (case 10, 33%), precluding within‐group comparison.

When asked to select one or more therapy options, dentists most frequently chose resin infiltration (70%) as part of the treatment plan, regardless of the indication or the correct diagnosis. Bleaching was chosen as a treatment option in approximately one third of all cases. Notably, the selection of bleaching was significantly influenced by diagnostic accuracy: it was more likely to be chosen for incorrectly diagnosed MIH and WSL cases but less likely for correctly diagnosed fluorosis cases. Other treatment choices were also significantly affected by diagnostic accuracy, including remineralization and composite restoration for fluorosis and the mixed case, as well as the decision to provide no treatment for WSL and fluorosis. Remineralization was strongly recommended for WSL, but less so for other diagnoses. Among all treatment options, veneers and crowns were the least frequently recommended.

Participants expressed confidence in their diagnosis in 72% of cases, with nearly all also demonstrating confidence in their treatment. Overall, 65% felt confident in both diagnosis and treatment. However, only 39% of all cases involved participants who were both confident and correct (Table 3). Multivariable analysis showed that diagnostic confidence and, in MIH cases, geographic location were the only factors significantly associated with diagnostic accuracy, with South American participants more likely to correctly diagnose MIH than Europeans (Table 4).

**TABLE 3:** Dentists' confidence in their diagnosis and treatment choice.

Diagnosis	Correct diagnosis	Confidence			Confidence in treatment and diagnosis and correct diagnosis
		In diagnosis	In treatment	In treatment and diagnosis	
WSL	50	76	73	68	40
Fluorosis	66	73	70	68	49
MIH	49	68	65	62	34
Mixed	33	73	71	66	24

**TABLE 4:** Factors influencing correct diagnosis, stratified by diagnoses.

Factors		WSL	Fluorosis	MIH	Mixed
Sex (ref: female)		0.7 (0.4–1.4)	0.7 (0.5–1.1)	0.7 (0.5–1.0)	0.8 (0.4–1.8)
Age (years)		0.9 (0.5–1.6)	1.1 (0.8–1.7)	0.8 (0.6–1.2)	1.2 (0.6–2.3)
Diagnostic confidence (ref: not confident)		3.3 (1.7–6.4)	3.3 (2.1–5.2)	2.0 (1.4–3.0)	1.3 (0.6–2.8)
Location (ref: Europe)	South America	1.0 (0.5–1.8)	0.9 (0.6–1.4)	1.5 (1.0–2.1)	0.8 (0.4–1.7)
	North America	1.8 (0.2–16.3)	3.0 (0.3–26.7)	1.1 (0.3–4.2)	6.3 (0.5–83.3)
	Middle East and Africa	0.5 (0.1–4.1)	1.1 (0.2–4.6)	1.6 (0.4–6.1)	0.7 (0.5–9.1)
Experience (ref: < 5 years)	5–10 years	1.1 (0.5–2.5)	0.8 (0.4–1.5)	1.2 (0.7–2.0)	0.5 (0.2–1.4)
	11–20 years	1.1 (0.4–3.4)	0.6 (0.3–1.4)	1.7 (0.8–3.4)	0.6 (0.2–2.3)
	21–30 years	0.8 (0.2–3.4)	0.7 (0.3–2.1)	1.8 (0.7–4.5)	0.4 (0.1–2.3)
	> 30 years	1.0 (0.1–7.4)	0.7 (0.2–3.0)	2.5 (0.7–8.7)	0.8 (0.1–9.0)
Employment (ref: employed at praxis or clinic)	Employed at university	1.4 (0.7–3.2)	1.5 (0.9–2.7)	0.9 (0.6–1.5)	0.6 (0.2–1.5)
	Self‐employed	0.8 (0.3–1.9)	0.7 (0.4–1.4)	0.9 (0.5–1.5)	1.4 (0.5–3.8)
	Other	1.0 (0.4–2.5)	1.0 (0.5–1.8)	0.8 (0.4–1.4)	1.1 (0.1–3.2)

*Note:* Odds ratios and their 95% CI are shown, with statistically significant differences being highlighted in gray (*p* < 0.05).

In contrast, factors associated with treatment preferences showed more heterogeneous patterns (Tables S2–S5). For WSL, selecting no treatment or bleaching was significantly influenced by diagnostic accuracy. Microinvasive treatment was associated with younger participants who nonetheless had more than 10 years of work experience and with those located in Europe. For fluorosis, treatment decisions were primarily driven by diagnostic accuracy: clinicians who made a correct diagnosis were more likely to choose no treatment or bleaching, whereas incorrect diagnoses more often led to recommendations for noninvasive or composite treatment. MIH treatment choices were strongly associated with geographic region, with clinicians in Europe more likely to recommend minimally invasive approaches. For the mixed case, although some variables were statistically associated with treatment choices, no consistent or interpretable pattern emerged.

## 4 Discussion

Based on the data presented in this study, diagnosing vestibular enamel defects and selecting appropriate individualized treatment strategies remain challenging for dental practitioners. Only 52% of cases were correctly diagnosed, consistent with findings from previous studies assessing different types of vestibular defects [20]. In this study, participants were provided solely with frontal images, thus lacking essential diagnostic information such as molar involvement or orthodontic history. Nevertheless, this situation is becoming increasingly prevalent in clinical practice, as the growing availability of digital tools has substantially facilitated the exchange of clinical records. This enables patients to seek diagnostic assessments and treatment advice (tele‐dentistry), while also allowing clinicians to obtain diagnostic input, discuss treatment options, and compare therapeutic strategies more efficiently [34, 35].

In this survey, despite the absence of comprehensive information, participants expressed considerable confidence in their diagnoses, with 72% reporting high certainty. These findings may indicate overconfidence or gaps in training or awareness among dental professionals regarding vestibular enamel defects. Nevertheless, regression analysis demonstrated a positive association between diagnostic confidence and accuracy (Table 4). While the esthetic management of vestibular enamel defects may be regarded as a specialized domain within dentistry, both general and pediatric dentists routinely encounter and manage these conditions. Because many vestibular enamel defects manifest early in childhood and the first esthetic concerns typically arise around the onset of adolescence, treatment concepts should prioritize preservation of hard tissue and therefore favor conservative approaches [13, 36, 37]. Noninvasive management is particularly affected by accurate diagnosis, as inappropriate interventions, such as applying “topical fluoridation” in cases of fluorosis or providing “no treatment” for carious lesions, may provide no benefit and may even worsen the condition. Furthermore, the reported treatment preferences support the strong emphasis on minimally invasive approaches and the growing acceptance of contemporary modalities such as resin infiltration, with only a small portion of participants selecting veneers or crowns as their preferred treatment option (Table 5).

**TABLE 5:** Treatment preference.

Dentists' diagnosis		Treatment preference						
		No treatment	Noninvasive (remineralization)	Bleaching	Microinvasive (resin infiltration)	Composite	Veneer	Crowns
WSL	Correct	7	57	14	75	21	3	0
	Incorrect	13	50	33	79	24	4	0
Fluorosis	Correct	27	14	49	64	30	12	1
	Incorrect	15	39	38	67	41	13	2
MIH	Correct	18	16	31	73	59	10	1
	Incorrect	19	16	45	69	52	16	1
Mixed	Correct	5	31	36	73	89	4	0
	Incorrect	9	9	33	73	66	3	1

*Note:* Values represent the percentage (%) of cases in which dentists recommended the specified treatment as part of therapy, categorized by correct versus incorrect diagnosis.

Statistically significant differences between correct and incorrect diagnoses for a given treatment are highlighted in gray (*p* < 0.05).

Approximately one‐quarter of all WSL, fluorosis, and MIH cases were misclassified as mixed defects, whereas the mixed case, which truly represented both MIH and enamel hypoplasia, was identified as MIH in 50% of responses (Table 2). This pattern suggests that although the primary condition was often recognized, additional features resembling other defects led clinicians to assume the presence of multiple conditions. This underscores the complexity of clinical presentations and the impact of overlapping features on diagnostic accuracy. Improving diagnostic accuracy may therefore require targeted education [38]. One study demonstrated that a brief training (2‐h lecture) can sustainably enhance dentists' abilities to diagnose and differentiate DDE [28]. Another study similarly found that dentists who received formal training were more capable of correctly identifying the defects and were more likely to select appropriate management strategies [20]. Alternatively, artificial intelligence (AI)‐based models for detecting vestibular enamel defects in clinical photographs are currently being developed in order to support more reliable diagnostics. Several research groups have reported accuracies exceeding 90%, indicating promising diagnostic potential. However, most reported models have been trained for specific indications (e.g., WSL) and remain unable to identify a broader spectrum of vestibular enamel defects. Furthermore, considerations of practical applicability and clinical validity require further investigation before such systems can be feasibly implemented in routine dental practice [39, 40, 41].

Certainly, diagnosis based solely on (frontal) clinical images presents inherent limitations. Essential contextual information, such as anamnestic data, is absent. Furthermore, the clinical images in this study did not depict the entire maxillary arch, thereby failing to provide information regarding the molars, which are a vital component in the identification of MIH, for instance. However, this information is often not adequately considered or simply unavailable. Furthermore, intraoral photographs are widely used in tele‐dentistry to support diagnosis, treatment planning, and the exchange of information within the professional network, which reflects the scenario this study sought to simulate [42]. It must be emphasized that, aside from the clinical presentation, comprehensive treatment plans must always consider patients' symptoms and individual needs, which are factors that cannot be adequately conveyed through the study design employed here. Moreover, the term fluorosis was retained throughout the study to maintain consistency with expert reference diagnoses and common usage among clinicians. However, it should be recognized that similar diffuse enamel opacities/dysmineralizations may arise from different etiologies, and that a definitive fluoride‐related origin cannot always be established solely from the clinical appearance. Future studies may incorporate concise case reports alongside the images to provide more comprehensive and insightful information to the participants and, consequently, yield more meaningful data. Still, the data presented in this study indicate a strong emphasis on conservative treatment options, with most participants preferring non‐ and microinvasive treatment options rather than veneers or crowns as discussed above. Another limitation of this cross‐sectional pilot survey is the small sample size of 10 clinical images. Because the clinical presentation of vestibular defects can vary considerably, not only by defect type but also by severity, a broader range of cases would be necessary to generate more robust data, particularly for selecting appropriate treatment options.

## 5 Conclusion

Dentists' accuracy in identifying various vestibular enamel defects on clinical images was limited, yet they often exhibited overconfidence in their diagnostic capabilities. Most dentists opted for a conservative approach, combining primarily noninvasive and microinvasive treatment modalities to manage the clinical conditions presented.

## Funding

The authors have nothing to report.

## Ethics Statement

Ethical approval for this study was granted by the institutional ethical review board of the Charité University on June 16, 2022 (EA4/069/22).

## Conflicts of Interest

The authors declare no conflicts of interest.

## Supporting information


**Table S1:** Per‐class diagnostic accuracy.
**Table S2:** Factors influencing treatment preference for WSL cases.
**Table S3:** Factors influencing treatment preference for fluorosis cases.
**Table S4:** Factors influencing treatment preference for MIH cases.
**Table S5:** Factors influencing treatment preference for the mixed case.

## Data Availability

The data that support the findings of this study are available on request from the corresponding author. The data are not publicly available due to privacy or ethical restrictions.
