# Supplementary material for: Diagnosis and Dentists' Treatment Preferences for Vestibular Enamel Defects—A Cross‐Sectional Survey
Source: J Esthet Restor Dent. 2026 Jun 22;38(11):2124–32. doi: 10.1111/jerd.70217 (PMC13680784; doi:10.1111/jerd.70217)
Supplement: Supplementary file 1 [file JERD-38-2124-s001.docx]

**Supplementary Information**

**Table S1** | Per-class diagnostic accuracy

| **Dentists’**  **diagnosis** | **Reference Diagnosis** | | | |
| --- | --- | --- | --- | --- |
|  | **Sensitivity** | **Specificity** | **PPV** | **NPV** |
| **WSL** | 50 | 98 | 86 | 89 |
| **Fluorosis** | 67 | 77 | 56 | 84 |
| **MIH** | 49 | 86 | 68 | 71 |
| **Mixed** | 33 | 77 | 14 | 91 |
| PPV: Positive Predictive Value; NPV: Negative Predictive Value  Values represent the percentages (%). | | | | |

**Table S2** | Factors influencing treatment preference for WSL cases.

| **WSL** | | | | | | | |
| --- | --- | --- | --- | --- | --- | --- | --- |
| **Factors** | | **No Treatment** | **Non-Invasive** | **Bleaching** | **Micro-invasive** | **Composite** | **Veneer** |
| **Diagnosis (ref: incorrect)** | | 0.5 (0.2–1.0) | 1.5 (0.9–2.4) | 0.3 (0.2–0.5) | 0.6 (0.3–1.1) | 0.8 (0.5–1.5) | 0.6 (0.2–2.3) |
| **Sex (ref: female)** | | 1.9 (0.9–4.2) | 0.7 (0.5–1.3) | 1.4 (0.8–2.4) | 1.3 (0.7–2.5) | 0.9 (0.5–1.7) | 1.0 (0.2–4.1) |
| **Age (years)** | | 1.5 (0.7–3.1) | 1.8 (1.1–2.8) | 0.6 (0.4–1.1) | 0.4 (0.2–0.8) | 1.5 (0.9–2.7) | 1.1 (0.3–4.2) |
| **Confidence in**  **treatment choice**  **(ref: not confident)** | | 1.0 (0.4–2.3) | 0.7 (0.4–1.2) | 1.4 (0.7–2.4) | 2.8 (1.4–5.8) | 1.2 (0.6–2-3) | 2.1 (0.4–11.0) |
| **Location**  **(ref: Europe)** | **South America** | 0.7 (0.3–1.7) | 1.7 (1.0–2.8) | 0.5 (0.3–1.0) | 0.1 (0.1–0.2) | 0.3 (0.1–0.6) | 1.5 (0.4–6.3) |
|  | **North America** | 2.8 (0.2–34.9) | - | 0.4 (0.1–3.7) | 0.1 (0.0–1.4) | 1.8 (0.2–18.7) | - |
|  | **Middle East & Africa** | - | 2.7 (0.4–16.9) | 1.2 (0.2–7.9) | 0.3 (0.0–3.3) | 0.3 (0.0–3.1) | - |
| **Experience**  **(ref: < 5)**  **In years** | **5 – 10** | 1.5 (0.5–4.5) | 2.0 (1.0–3.9) | 1.6 (0.7–3.7) | 1.2 (0.5–3.1) | 0.8 (0.4–1.9) | 0.5 (0.1–2.8) |
|  | **11 – 20** | 0.2 (0.0–1.4) | 0.5 (0.2–1.3) | 2.7 (0.9–8.6) | 7.0 (1.8–26.8) | 0.3 (0.1–1.1) | 0.5 (0.1–5.6) |
|  | **21 – 30** | 0.3 (0.0–2.4) | 0.6 (0.2–2.0) | 3.5 (0.8–15.7) | 5.4 (1.2–25.1) | 0.3 (0.1–1.5) | 0.2 (0.0–7.8) |
|  | **> 30** | 0.1 (0.0–1.6) | 0.2 (0.0–1.2) | 8.4 (1.1–66.5) | 6.6 (0.8–56.0) | 0.3 (0.0–2.4) | - |
| **Employment (ref: employed at praxis or clinic)** | **University** | 1.6 (0.6–4.5) | 0.8 (0.4–1.6) | 0.8 (0.4–1.7) | 2.0 (0.8–5.3) | 0.3 (0.1–0.8) | - |
|  | **Self-employed** | 0.8 (0.2–3.0) | 0.8 (0.4–1.7) | 0.5 (0.2–1.1) | 1.1 (0.4–2.9) | 1.5 (0.6–3.5) | 0.2 (0.0–1.9) |
|  | **Other** | 2.3 (0.7–7.3) | 1.2 (0.6–2.4) | 0.7 (0.3–1.6) | 0.7 (0.3–1.6) | 2.5 (1.1–5.7) | 0.9 (0.2–3.6) |
| Odds ratios and their 95% CI are shown, with statistically significant differences being highlighted in grey (p < 0.05). | | | | | | | |

**Table S3** | Factors influencing treatment preference for fluorosis cases.

| **Fluorosis** | | | | | | | |
| --- | --- | --- | --- | --- | --- | --- | --- |
| **Factors** | | **No Treatment** | **Non-Invasive** | **Bleaching** | **Micro-invasive** | **Composite** | **Veneer** |
| **Diagnosis (ref: incorrect)** | | 2.0 (1.2–3.3) | 0.3 (0.2–0.4) | 1.5 (1.0–2.3) | 0.8 (0.5–1.2) | 0.7 (0.4–1.0) | 1.0 (0.6–1.8) |
| **Sex (ref: female)** | | 0.9 (0.6–1.5) | 1.0 (0.6–1.7) | 1.0 (0.7–1.5) | 1.5 (1.0–2.2) | 1.0 (0.7–1.6) | 1.0 (0.6–1.8) |
| **Age (years)** | | 1.5 (1.0–2.3) | 1.5 (1.0–2.4) | 0.8 (0.6–1.1) | 0.9 (0.6–1.3) | 1.2 (0.9–1.8) | 1.0 (0.6–1.8) |
| **Confidence in**  **treatment choice**  **(ref: not confident)** | | 2.0 (1.2–3.5) | 0.7 (0.4–1.2) | 1.1 (0.7–1.7) | 1.6 (1.0–2.4) | 0.8 (0.5–1.2) | 0.6 (0.3-1.1) |
| **Location**  **(ref: Europe)** | **South America** | 0.5 (0.3–0.9) | 0.6 (0.3–1.0) | 1.0 (0.7–1.5) | 0.7 (0.5–1.0) | 0.9 (0.6–1.3) | 0.5 (0.3–1.1) |
|  | **North America** | 0.5 (0.1–4.1) | - | 0.5 (0.1–2.0) | - | 0.3 (0.0–2.5) | - |
|  | **Middle East & Africa** | 0.5 (0.1–2.7) | 1.2 (0.3–5.8) | 4.6 (1.0–20.3) | 1.0 (0.2–4.5) | 0.3 (0.0–2.4) | - |
| **Experience**  **(ref: < 5)**  **In years** | **5 – 10** | 1.2 (0.6–2.4) | 2.2 (1.0–4.9) | 0.8 (0.5–1.5) | 0.6 (0.3–1.0) | 0.8 (0.5–1.5) | 0.8 (0.3–1.6) |
|  | **11 – 20** | 0.2 (0.1–0.6) | 1.3 (0.5–3.6) | 1.9 (0.9–4.1) | 1.3 (0.6–3.0) | 0.7 (0.4–1.6) | 0.5 (0.2–1.5) |
|  | **21 – 30** | 0.4 (0.1–1.4) | 1.1 (0.3–3.9) | 2.4 (0.9–6.4) | 0.7 (0.2–1.8) | 0.5 (0.2–1.5) | 0.7 (0.2–3.0) |
|  | **> 30** | 0.2 (0.0–1.2) | 0.7 (0.1–4.1) | 1.2 (0.3–4.7) | 0.6 (0.1–2.6) | 0.4 (0.1–1.8) | 0.5 (0.1–4.3) |
| **Employment (ref: employed at praxis or clinic)** | **University** | 1.7 (0.9–3.2) | 1.2 (0.6–2.5) | 0.7 (0.4–1.2) | 1.2 (0.7–2.1) | 0.7 (0.4–1.2) | 1.6 (0.7–3.6) |
|  | **Self-employed** | 1.5 (0.7–2.9) | 1.7 (0.8–3.5) | 0.5 (0.3–0.9) | 1.1 (0.6–2.0) | 0.7 (0.4–1.4) | 1.4 (0.6–3.7) |
|  | **Other** | 1.2 (0.6–2.5) | 1.7 (0.8–3.5) | 0.6 (0.3–1.1) | 1.3 (0.7–2.4) | 0.9 (0.5–1.6) | 4.1 (1.8–9.5) |
| Odds ratios and their 95% CI are shown, with statistically significant differences being highlighted in grey (p < 0.05). | | | | | | | |

**Table S4** | Factors influencing treatment preference for MIH cases.

| **MIH** | | | | | | | |
| --- | --- | --- | --- | --- | --- | --- | --- |
| **Factors** | | **No Treatment** | **Non-Invasive** | **Bleaching** | **Micro-invasive** | **Composite** | **Veneer** |
| **Diagnosis (ref: incorrect)** | | 1.0 (0.7–1.5) | 1.2 (0.8–1.9) | 0.5 (0.4–0.7) | 1.1 (0.8–1.6) | 1.4 (1.0–1.9) | 0.7 (0.4–1.1) |
| **Sex (ref: female)** | | 0.7 (0.4–1.1) | 1.1 (0.7–1.8) | 1.2 (0.9–1.7) | 0.9 (0.6–1.4) | 1.2 (0.8–1.6) | 1.3 (0.8–2.1) |
| **Age (years)** | | 1.0 (0.7–1.5) | 2.1 (1.4–3.3) | 0.7 (0.5–1.0) | 0.8 (0.5–1.1) | 1.0 (0.8–1.4) | 0.9 (0.5–1.4) |
| **Confidence in**  **treatment choice**  **(ref: not confident)** | | 1.0 (0.7–1.6) | 0.4 (0.3–0.7) | 1.2 (0.8–1.7) | 3.2 (2.2–4.8) | 1.5 (1.1–2.2) | 0.7 (0.4–1.1) |
| **Location**  **(ref: Europe)** | **South America** | 0.5 (0.3–0.9) | 0.4 (0.3–0.7) | 1.0 (0.7–1.5) | 0.5 (0.4–0.8) | 0.6 (0.4–0.9) | 1.3 (0.8–2.2) |
|  | **North America** | 3.4 (0.9–13.0) | - | 0.1 (0.0–0.9) | 0.4 (0.1–1.4) | 0.7 (0.2–2.4) | - |
|  | **Middle East & Africa** | 0.7 (0.4–3.1) | 1.5 (0.4–5.9) | 1.8 (0.5–6.2) | 0.5 (0.2–1.8) | 0.1 (0.0–0.5) | - |
| **Experience**  **(ref: < 5)**  **In years** | **5 – 10** | 2.6 (1.4–5.0) | 2.7 (1.1–6.5) | 1.0 (0.6–1.7) | 0.5 (0.3–0.9) | 1.0 (0.6–1.6) | 0.8 (0.4–1.5) |
|  | **11 – 20** | 0.9 (0.3–2.2) | 1.0 (0.3–2.8) | 2.0 (1.0–3.9) | 1.0 (0.5–2.2) | 0.8 (0.4–1.6) | 1.1 (0.5–2.9) |
|  | **21 – 30** | 1.3 (0.4–4.1) | 0.8 (0.2–2.9) | 3.6 (1.5–8.7) | 1.2 (0.4–3.1) | 1.2 (0.5–2.8) | 0.6 (0.2–2.2) |
|  | **> 30** | 0.9 (0.2–4.1) | 0.9 (0.2–5.1) | 2.6 (0.7–9.2) | 0.8 (0.2–3.1) | 1.1 (0.3–3.8) | 0.2 (0.0–1.9) |
| **Employment (ref: employed at praxis or clinic)** | **University** | 1.4 (0.8–2.4) | 0.9 (0.5–1.8) | 0.9 (0.6–1.4) | 0.9 (0.5–1.5) | 0.9 (0.6–1.4) | 1.5 (0.8–3.0) |
|  | **Self-employed** | 1.6 (0.9–3.1) | 1.4 (0.7–2.8) | 0.6 (0.4–1.0) | 0.5 (0.3–0.8) | 0.9 (0.6–1.5) | 1.4 (0.7–3.1) |
|  | **Other** | 0.9 (0.4–1.7) | 1.4 (0.7–3.0) | 1.0 (0.6–1.6) | 0.7 (0.4–1.3) | 1.3 (0.8–2.2) | 1.9 (0.9–3.9) |
| Odds ratios and their 95% CI are shown, with statistically significant differences being highlighted in grey (p < 0.05). | | | | | | | |

**Table S5** | Factors influencing treatment preference for the mixed case.

| **Mixed** | | | | | | | |
| --- | --- | --- | --- | --- | --- | --- | --- |
| **Factors** | | **No Treatment** | **Non-Invasive** | **Bleaching** | **Micro-invasive** | **Composite** | **Veneer** |
| **Diagnosis (ref: incorrect)** | | 0.5 (0.1–2.1) | 5.7 (2.1–15.4) | 1.1 (0.5–2.4) | 1.0 (0.5–2.4) | 4.1 (1.5–11.0) | 0.3 (0.0–3.7) |
| **Sex (ref: female)** | | 2.3 (0.6–8.3) | 1.3 (0.5–3.5) | 0.8 (0.4–1.6) | 1.2 (0.5–2.6) | 1.0 (0.5–2.3) | 0.2 (0.0–4.2) |
| **Age (years)** | | 1.0 (0.4–3.1) | 1.3 (0.5–3.3) | 0.7 (0.3–1.3) | 0.6 (0.3–1.3) | 1.0 (0.5–2.2) | 1.0 (0.0–32.2) |
| **Confidence in**  **treatment choice**  **(ref: not confident)** | | 0.9 (0.2–3.5) | 0.6 (0.2–1.7) | 1.6 (0.7–3.7) | 2.1 (0.9–4.9) | 1.8 (0.8-4.3) | - |
| **Location**  **(ref: Europe)** | **South America** | 0.8 (0.2–3.2) | 0.9 (0.3–2.3) | 0.5 (0.2–1.0) | 0.8 (0.4–1.8) | 0.4 (0.2–0.9) | 0.3 (0.0–4.2) |
|  | **North America** | 14.1 (0.6–316.9) | - | - | - | 0.2 (0.0–2.6) | - |
|  | **Middle East & Africa** | - | - | 0.7 (0.1–9.6) | 0.2 (0.0–2.6) | 0.5 (0.0–7.5) | - |
| **Experience**  **(ref: < 5)**  **In years** | **5 – 10** | 1.9 (0.3–12.3) | 2.1 (0.5–10.0) | 0.7 (0.2–2.0) | 0.6 (0.2–1.9) | 0.4 (0.1–1.4) | - |
|  | **11 – 20** | 0.5 (0.0–8.1) | 0.7 (0.1–5.8) | 1.6 (0.4–6.4) | 2.4 (0.4–12.6) | 0.5 (0.1–2.6) | 0.1 (0.0–15.0) |
|  | **21 – 30** | 1.3 (0.1–30.1) | 1.7 (0.1–22.9) | 1.8 (0.3–12.2) | 1.5 (0.2–11.0) | 0.3 (0.0–2.7) | 0.3 (0.0–373.8) |
|  | **> 30** | 0.5 (0.0–38.5) | 1.7 (0.1–56.8) | 4.9 (0.3–70.9) | 3.0 (0.2–51.6) | 0.5 (0.0–8.6) | - |
| **Employment (ref: employed at praxis or clinic)** | **University** | 1.4 (0.2–9.6) | 3.5 (0.6–20.3) | 0.6 (0.2–1.4) | 0.5 (0.1–1.5) | 2.3 (0.8–7.2) | 0.1 (0.0–2.0) |
|  | **Self-employed** | 2.5 (0.3–18.2) | 5.1 (0.8–30.9) | 0.5 (0.2–1.4) | 0.3 (0.1–1.2) | 1.3 (0.4–4.3) | 0.1 (0.0–1.3) |
|  | **Other** | 3.1 (0.4–23.2) | 5.6 (0.9–34.9) | 0.2 (0.1–0.8) | 0.4 (0.1–1.5) | 1.7 (0.5–5.7) | - |
| Odds ratios and their 95% CI are shown, with statistically significant differences being highlighted in grey (p < 0.05). | | | | | | | |
